# Minimal change in children’s lifestyle behaviours and adiposity following a home-based obesity intervention: results from a pilot study

**DOI:** 10.1186/s13104-015-1796-2

**Published:** 2016-01-13

**Authors:** Nicola J. Spurrier, Lucinda Bell, Annabelle Wilson, Elizabeth Lowe, Rebecca Golley, Anthea A. Magarey

**Affiliations:** Department of Public Health, University of Adelaide, Adelaide, 5000 Australia; Department of Paediatrics and Child Health, Flinders University, Bedford Park, 5042 Australia; Department for Health and Ageing, SA Health, Citi Centre Building, 11 Hindmarsh Square, Adelaide, SA 5000 Australia; Nutrition and Dietetics, Flinders University, Bedford Park, 5042 Australia; Department of Public Health, Flinders University, Bedford Park, 5042 Australia; Division of Health Sciences City East Campus, University of South Australia, Adelaide, 5001 Australia; Sansom Institute for Health Research, University of South Australia, Adelaide, 5001 Australia

**Keywords:** Child, Obesity, Treatment, Home environment, Diet, Family

## Abstract

**Background/Methods:**

Families of overweight and obese children require support to make sustainable lifestyle changes to improve their child’s diet and activity behaviours and in turn weight status. The aim of this pre-post intervention pilot study was to evaluate the feasibility of an individualised home-based intervention for treatment seeking overweight/obese 4–12 year olds and their caregivers. Baseline measures were used to develop a family-specific intervention to improve the quality of the home environment. The intervention was delivered as individualised written recommendations and resources plus phone call and home visit support. Baseline measures were repeated approximately 6 months later.

**Results:**

Complete data for 24 children was available. Parents reported that 43 % of intervention recommendations were implemented ‘very much’. Some descriptive changes were observed in the home environment, most commonly including fruit and vegetables in their child’s lunchbox, not providing food treats, and restricting children’s access to chips/savoury snack biscuits. At the group level, minimal change was detected in children’s diet and activity behaviours or weight status (all p > 0.05).

**Conclusion:**

The study findings did not support intervention feasibility in its current form. Future interventions should target the family food and activity environment, but also utilise an approach to address the complex social circumstances which limit parent’s ability to prioritise healthy family lifestyle behaviours.

Trial registration: Australian New Zealand Clinical Trials Registry (ANZCTR) 3/12/2014. http://www.ANZCTR.org.au. ACTRN12614001264673

## Background

Childhood obesity is recognised as one of the most significant public health problems in developed countries. Families of overweight children need services and support to make sustainable lifestyle changes to improve their child’s diet and activity behaviours and in turn weight status [1, 2].

The obesity epidemic is largely considered to be due to increased availability of energy-dense foods and reduced opportunities for physical activity, characteristics of a modern society [3, 4]. Obesity-promoting environments have been described in terms of macro- and micro- environments [4]. The family home is an important micro-environment with the potential to influence children’s lifestyle behaviours and weight status [5]. Observational studies have identified modifiable characteristics of children’s home food and activity environments that are associated with diet quality and activity levels [6–10]. For example, we have previously characterised the home environment of 208 preschool children [11]. Parental role-modelling of physical activity, a safe and engaging backyard, having rules for use of small screen entertainment, using appropriate child feeding behaviours, and stocking more healthy food and less energy-dense nutrient poor foods in the home were associated with greater physical activity levels, less sedentary behaviour and healthier dietary patterns [11].

Child weight management that is based on practical advice on how to create a supportive home environment may be more effective than prescribing strict dietary or physical activity regimens, particularly if changes can become incorporated into a daily routine and sustained long term [12, 13]. Strategies to improve the home environment are often provided as part of weight management advice in clinic settings. However it can be difficult for families to translate this advice into reality. Working with parents in their homes is a potential alternative intervention setting which may make it easier for health care providers to assess areas for change and better appreciate family barriers. Home visits may also facilitate development of supportive therapeutic relationships. Home visiting has been trialled in obesity prevention studies with young children [14], but to date this approach has not been used in obesity treatment interventions for children who are already overweight or obese.

The home has been shown to be a successful intervention setting in other health areas, the best example being home visiting by nurses or other trained health visitors to support mothers living in disadvantage during the early years of child rearing [15]. Other examples in paediatrics include chronic diseases such as asthma [16, 17]. Some of the postulated reasons for the success of such programs include the increased ability for health visitors to build successful therapeutic relationships with family members and the fact that health visitors can more fully appreciate the socioeconomic and cultural environment in which families operate.

The aim of the current pilot intervention study was to assess the feasibility of an individualised home-based intervention for children with obesity whose parents were seeking treatment. Outcomes assessed included changes to the home environment and improvements in children’s dietary and physical activity behaviours and weight status.

## Methods

### Study design and recruitment

A pre-post design evaluated a home-based lifestyle support intervention conducted in 2008. It was hypothesized that an individualised home-based intervention would be a feasible child obesity treatment approach. Children aged 4–12 years who were overweight or obese according to the International Obesity Task Force (IOTF) Body Mass Index (BMI) cut-points [18] and who were living in metropolitan Adelaide were recruited via referrals to the Department of Paediatrics and Child Health, Flinders Medical Centre for management of obesity. Exclusion criteria were medical conditions affecting weight or growth or being enrolled in any other structured weight management program, however no child was excluded on these grounds. Children could continue to be managed by their general practitioner or paediatrician during the intervention. Written informed parent consent and child assent was obtained. The study was approved by the Flinders University Social and Behavioural Research Ethics committee.

**Fig. 1:**
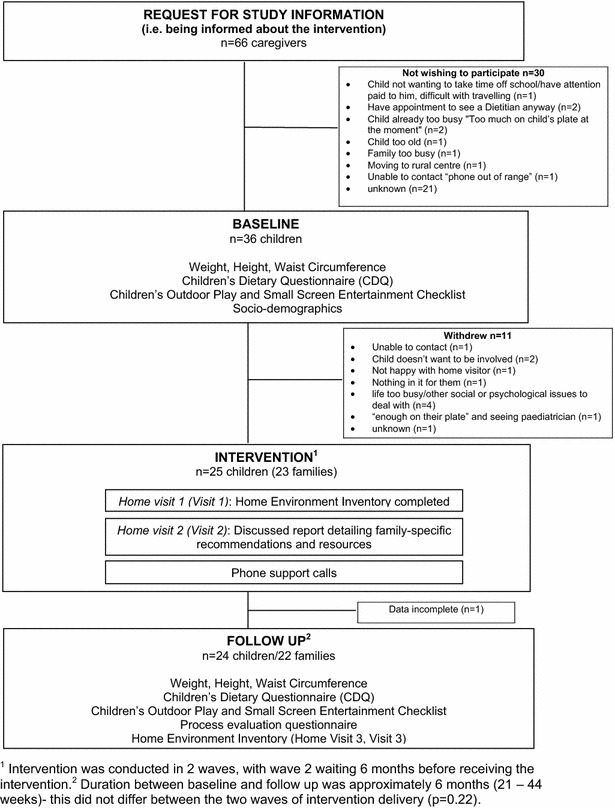
Flow of participants and data collection timing

The study sequence is shown in Fig. 1. At baseline and follow up, participants attended a 1-h assessment at which anthropometry and questionnaire data were obtained. This was followed by a 75-min home visit to assess the home environment. The three home visitors were trained via a 3 h workshop covering the socioecological basis of childhood obesity, dietary and physical activity guidelines, and how to work with families in non-judgemental partnerships. Comprehensive written instructions and a training site visit ensured a standardised procedure was followed.

### Measures

At baseline a socio-demographic questionnaire was completed by parents, and child weight, height and waist circumference were measured to the nearest 0.1 kg and 0.1 cm, using standardised scales and stadiometer. These measurements were undertaken in the paediatric outpatient department, Flinders Medical Centre, using the same set of scales and stadiometer for each child and at both baseline and follow-up. The research assistant undertaking these measurements was not involved in the home visit intervention and had been trained in anthropometry. Specifically, children were weighed on portable digital scales wearing light clothing, with shoes and socks removed. Height was measured using a wall-mounted stadiometer with shoes and socks removed, heels touching the wall and head in the ‘Frankfort’ plane. Waist circumference was measured using a standard metric tape measure held horizontally at a level midway between the lower rib margin and iliac crest (approximately in line with the umbilicus). BMI was calculated (weight, kg/height, m^2^) and weight status determined by applying the IOTF cut-points [18]. BMI and waist circumference z-scores were calculated using LMSGrowth Excel add-in which is based on 1990 United Kingdom reference data [19, 20].

Parents completed the 24-item Children’s Dietary Questionnaire (CDQ) [21] which generates four continuous scores (i.e. fruit and vegetables, non-core foods, fat from dairy, sweetened beverages). Non-core foods are defined as foods that are not essential to meet nutrient requirements and contain excess energy, fat, sugar and salt [22]. The CDQ shows acceptable reliability (ICC 0.5–0.9) and relative validity at the group level for the fruit and vegetables and non-core food scores (Cronbach alpha 0.62–0.72) [21].

Child activity patterns were measured using an adapted version of The Outdoor Play and Small Screen Entertainment Checklist [11, 23]. Parents reported the average time children spent per weekday and weekend day (in hours and minutes) (1) playing outdoors, (2) doing moderate-vigorous physical activity, and (3) using electronic media such as television and computers for both. Six items were added to the original checklist to explore average time spent per day (4) participating in physical activity and (5) using small screen entertainment. Times were averaged (weighted for type of day) to calculate (1) daily activity (vigorous physical activity + outdoor play) and (2) daily sedentary activity.

The Home Environment Inventory provides an assessment of 70+ family home characteristics associated with children’s dietary, physical activity, or sedentary behaviours [11]. Items cover the outdoor environment to support children’s play, parental role modelling, accessibility of small screen entertainment, parent behaviours around food preparation and availability of healthy and non-core foods and beverages in the home and child’s lunchbox. Inventory items were assessed by either direct observation or by parental report (details described elsewhere [11]).

### Study intervention

**Fig. 2:**
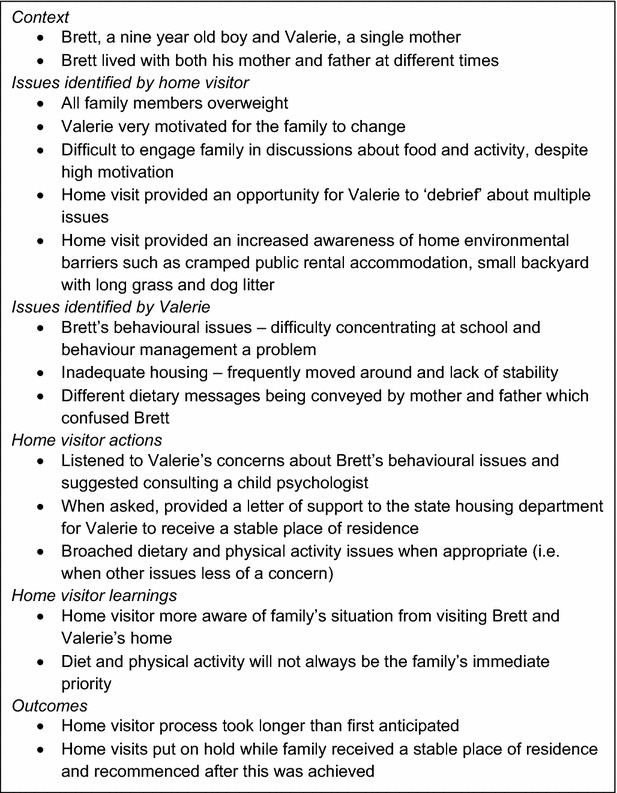
Intervention case study—Brett and Valerie

The study intervention was based on ecological theory [3, 24, 25]. The intervention was undertaken in family’s homes by trained visitors. Three home visits and two follow-up phone calls were offered to each family. Three home visitors were trained in the methodology with particular emphasis on being nonjudgmental, supportive and sensitive to socioeconomic determinants of obesity. Home visitors were chosen for their interpersonal skills and in this study all three were in the process of completing bachelor degrees in either nutrition, occupational therapy or human movement. The family was contacted by telephone by the home visitor who introduced themselves, explained the process and made a suitable time for the first home visit (Visit 1). At Visit 1 the home visitor clarified that the carer (in all instances this was the mother) understood the study and evaluated the family’s home environment using the Home Environment Inventory. The completed Home Environment Inventory was used to identify changes the family needed to make to improve the home environment. These changes were set as the intervention goals. To provide advice for each family, these goals were used to individualise a report template containing 25 strategies, along with ideas of how each strategy could be implemented. The ten most relevant strategies for each family were highlighted at the beginning of the report. This report was presented to the family at a second home visit (Visit 2), conducted approximately 2 weeks (±1 week) after Visit 1. The purpose of Visit 2 was to go through the report with the parent and to discuss ways of achieving change that the parent felt would be manageable for their family situation. The strategies were based on key evidence for improving healthy eating and physical activity in children [5, 22, 25–28]. Any additional issues relevant to achieving the desired outcomes that mother/family/carer identified during the home visit were noted for example housing, budgeting and child behaviour. The impact of these socioeconomic factors on the ability of families to institute change became more obvious once the study progressed and by the fact that visits were undertaken in the family home. The importance of this aspect of obesity management is illustrated in the case study provided in Fig. 2.

At Visit 2, time was made for the first follow-up phone call approximately 1 month after Visit 2. The purpose of this phone call was to gauge the family’s progress, provide ongoing support and encouragement with regards to the strategies and to provide further advice and support if requested. If families requested additional information, standard resources were posted to them (for example label reading) or a suggestion was provided about where they could access assistance (for example a child psychologist). The phone calls also provided opportunity to discuss any strategies that families felt were not working, and make alternative suggestions. At the end of the call, a time was made for the second follow-up phone call which was to occur approximately a month after the first phone call. And at the end of this call, a time for the final home visit (Visit 3) was made (aiming to be approximately 4 months after Visit 1). At Visit 3, the inventory was repeated. Encouragement for continuing to make lifestyle changes was also given to the families at this final visit.

### Statistical analysis

Analyses were conducted using SPSS for Windows version 17.0. Descriptive statistics are presented as frequencies or mean (standard deviation)/median (IQ range). Wilcoxin signed-rank tests and paired t tests were used to assess changes from baseline to follow up in subscale scores for the CDQ, Physical Activity Questionnaire and anthropometric data. Categorical data from the Home Environment Inventory were analysed using Chi square tests for independence. Statistical significance was set at p < 0.05.

## Results

### Sample characteristics

Figure 1 summarises the flow of participants through the study (duration approximately 6 months, 21–44 weeks). Complete follow-up data was available for 24 children (14 boys; 25 % overweight and 75 % obese). Demographic characteristics of study families are shown in Table 1.

**Table 1:** Demographic characteristics of study participants at baseline

Characteristic	N (%) or mean (SD)
Child’s age (years) n = 24	7.8 (±2.3)
Child’s gender n = 24	Male:Female 14:10
Household structure (n = 22)	
Two parent household	15 (68.2)
Single parent household	7 (31.8)
Mother’s education level (n = 21)	
Some years of high school	2 (9.5)
Year 12, HSC or equivalent	5 (23.8)
Technical, trade, or TAFE certificate	5 (23.8)
Tertiary qualifications	9 (42.9)
Father’s education level (n = 22)	
Some years of high school	5 (22.7)
Year 12, HSC or equivalent	4 (18.2)
Technical, trade, or TAFE certificate	5 (22.7)
Tertiary qualifications	8 (36.4)
Mother in paid employment (n = 21)	
Yes	17 (81.0)
Father in paid employment (n = 22)	
Yes	20 (90.9)
Household income (per week) (n = 17)	
<$500	2 (11.8)
<$500–$999	3 (17.6)
$1000–$1499	3 (17.6)
$1500–$1999	4 (23.5)
≥$2000	5 (29.4)

### Home environment characteristics

Table 2 shows the most frequent recommendations made to families in the intervention phase. Forty-three percent of recommendations were implemented ‘very much’ (Table 2). Baseline and follow up family environment characteristics as measured at the home visit are shown in Table 3. Recommendations to include fruit and vegetables in their child’s lunchbox, not providing food treats, and restricting access to chips/savoury snack biscuits (in the home and lunchbox), appeared to be implemented more easily than other recommendations. Parents being active every day, children spending time outside, family members not eating in front of the television and restricting the availability of certain non-core foods in the home appeared more difficult to implement.

**Table 2:** Common intervention recommendations and caregiver-reported success in implementing change at home (n = 22 families)

Recommendation	Number of families given this recommendation	How well were changes implemented? (self-reported)			
		Very much	Some-what	A little	Not at all
Encourage all household members to spend some active time outside each day	21	9	7	5	0
Spend time outside with your child daily	19	8	5	5	1
Restrict availability of non-core foods (e.g. savoury snacks, cake)	16	8	7	1	0
Include fruit and vegetables in your child’s lunch box every day	15	11	3	0	1
Reduce portion sizes	12	5	6	1	0
Avoid family members eating in front of TV	9	3	3	1	2
Limit takeaway food to 1–2 times per month	9	5	4	0	0
Restrict sweetened beverages (including fruit-juice)	8	4	4	0	0
Try new strategies to avoid wasting food	8	5	2	1	0
Avoid having the TV on during mealtimes	7	3	0	1	3
Avoid putting non-core snack foods in your child’s lunch box (including muesli/health/breakfast bars)	7	2	4	1	0

*TV* television

**Table 3:** Frequency (percentage) of selected home environment inventory variables pre- and post-intervention (n = 22)

Variable		Response category	Baseline	Follow up
Parental role modelling of physical activity				
How often	Mother walks for ≥ 30 min	Once/month or more	18 (82)	15 (68)
	Father walks for ≥ 30 min	Once/month or more	14 (67)	18 (82)
How many	Hours of TV mother watches/day	>2 h	5 (26)	6 (30)
	Hours of TV father watches/day	>2 h	14 (70)	9 (47)
Opportunities for outdoor play				
How often	Child taken to the playground	≥Twice/week	3 (14)	4 (18)
	Child taken to the park	≥Twice/week	2 (9)	2 (9)
Family norms				
How often	Child eats evening meal in front of TV	Frequently/sometimes	10 (45)	10 (48)
	Other family members eat food in front TV	Frequently/sometimes	10 (45)	10 (45)
	Adults set rules for child’s TV viewing	Very much/quite a bit	12 (57)	15 (75)
	Adults limit child’s exposure to TV advertising	Very much/quite a bit	6 (27)	8 (38)
Food accessibility				
How often adults restrict child access to	Fruit juice	Frequently/Sometimes	16 (73)	16 (73)
	High fat/high sugar foods	Frequently/Sometimes	22 (100)	21 (95)
	Soft drink/cordial	Frequently/Sometimes	18 (82)	18 (82)
How often	Adults give child food ‘treats’	Frequently/Sometimes	9 (41)	5 (23)
What is	Portion size of child’s main meal	>1/2 average dinner plate	9 (41)	7 (32)
How family	Feels about wasted food	Very accepting/accepting	10 (45)	12 (54)
Availability of food in the home				
Amount of	Fruit	>3 kg	15 (68)	16 (73)
	Vegetables	>6 kg	13 (59)	12 (55)
	Fruit juice	>2L	5 (23)	7 (32)
	Dairy	All reduced fat	16 (73)	18 (82)
	Chips/savoury snack biscuits	>100 g	17 (77)	5 (68)
	Lollies/sweets/chocolate	>120 g	12 (55)	13 (59)
	Muesli/chocolate bars	>0.5 boxes	14 (64)	13 (59)
	Cakes/biscuits	>140 g	12 (55)	11 (50)
	Soft drink/cordial	>2L	18 (82)	15 (68)
Availability of food in the child’s lunchbox				
How often lunchbox contains	Fruit	Frequently/sometimes	20 (91)	21 (95)
	Vegetables	Frequently/sometimes	7 (32)	13 (59)
	Chips/savoury snack biscuits	Frequently/sometimes	12 (54)	7 (32)
	Lollies/sweets/chocolate	Frequently/sometimes	1 (5)	1 (5)
	Muesli/breakfast bars	Frequently/sometimes	7 (32)	7 (32)
	Cakes/biscuits	Frequently/sometimes	7 (32)	7 (32)

Variables presented reflect the most common areas for concern for families in the intervention

*TV* television

### Children’s lifestyle and anthropometry

Baseline and follow up lifestyle behaviours and anthropometry are shown in Table 4. Score improvements occurred in about half the sample for *fruit and vegetables* (n = 12), *sweetened beverages* (n = 14)*, non*-*core food* (n = 12), *outdoor playtime* (n = 14), *sedentary time* (n = 11) and *time spent in daily activity* (n = 13). Around one-third demonstrated improvements in *time spent in sedentary activity* (n = 9). One child shifted from “overweight” into the “healthy weight” range, all others remained in the same weight category. Improvements in BMI z scores were recorded for 15/24 children; 10 decreasing by ≥5 % and 5 decreasing by ≤5 %. Improvements in waist circumference z scores were recorded for 18 children; 12 decreasing by ≥5 % and 6 decreasing by ≤5 %. On the other hand, BMIz score increased by >5 % in 3/24 children and Waist Circumference z scores increased by >5 % in 3/24 children.

**Table 4:** Children’s anthropometry, dietary and activity scores pre- and post-intervention (n = 24)

	Baseline	Follow-up	*P* value
Anthropometric characteristics			
Height (cm)	135.2 ± 14.3	138.7 ± 13.8	
Weight (kg)	45.7 ± 13.8	48.6 ± 13.0	0.46
BMI (kg/m^2^)	24.5 ± 3.2	24.8 ± 3.1	0.77
BMI z score	2.9 ± 0.7	2.8 ± 0.7	0.69
Waist circumference (cm)	77.8 ± 9.7	77.7 ± 8.7	0.97
Waist circumference Z score	3.2 ± 0.6	3.0 ± 0.5	0.22
CDQ scores			
Fruit and vegetables	11.4 ± 4.4	10.9 ± 4.1	0.60
Fat from dairy	0.9 ± 0.9	1.0 ± 1.2	0.73
Sweetened Beverages	1.4 ± 1.1	1.3 ± 1.0	0.38
Non-core foods	2.0 ± 0.8	1.7 ± 0.6	0.79
Activity scores			
Outdoor playtime	6.7 ± 3.5	6.8 ± 3.3	0.56
Sedentary time	2.9 ± 1.9	2.7 ± 1.4	0.60
Minutes daily activity	163 ± 61	174 ± 109	0.93
Minutes sedentary activity	140 ± 74	132 ± 69	0.59

Duration between baseline (pre-intervention) and follow up (post-intervention) ~6 months

## Discussion

This pilot study explored the feasibility of a home-based child weight management intervention. Families from a range of different socioeconomic backgrounds participated in the study. The intervention involved direct assessment of the family food and activity environment which informed individualised recommendations for family change. It was hypothesised that improving the family food and activity environment would lead to improvements in children’s dietary and physical activity behaviours, and thus weight status. While the study findings support the family environment as a target for intervention, provision of simple individualised recommendations appeared to be insufficient to enable families to significantly alter their home food and activity environment.

Baseline findings demonstrated the potential for improvement in the home environments of this sample of overweight and obese children. For example, while it was common for families to eat together, so was eating meals in front of the television. Likewise, while healthy foods such as fruit and vegetables were available in homes, so were large amounts of energy dense, nutrient poor foods. In terms of physical activity, role modelling of television viewing was more common than modelling of activity and in general there were limited opportunities for active play.

The most frequent intervention recommendations made to families targeted parental role modelling of physical activity, encouragement of outdoor active play, reducing children’s exposure to energy dense nutrient poor foods and increasing availability of fruit and vegetables in lunchboxes. Post-intervention, small positive changes in the home environment were observed. Most related to children’s access to food in the home and lunchbox; greater access to vegetables and restriction of high fat/high salt non-core foods and food ‘treats’. Recommendations which appeared difficult for parents to implement were purchasing take-away food, eating food in front of the TV, having large amounts of non-core food in the home, and taking children to playgrounds or parks. Whilst some individual change occurred in lifestyle behaviours, at the group level few changes were detected in children’s diet and activity patterns. While BMI and Waist Circumference z scores improved for approximately two-thirds of children, at the group level the changes were small relative to the effects observed in other child weight management intervention studies [12] even accounting for the small sample size.

A home visit approach enables direct assessment of the home environment so that families can receive tailored advice about changes to improve children’s diet, activity patterns and weight status. However in this pilot study, families either did not achieve the necessary change or the degree of change was not sufficient to detect improvements in children’s diet and activity behaviours or weight status. The lack of intervention effect may reflect the need for more intensive and ongoing support particularly with respect to competing demands on family time and resources. Multidisciplinary expertise is often needed to appreciate and confront the complex psychosocial and environmental constraints families are challenged with, particularly those living in disadvantage. By this we mean that the skill-set of the team needs to include provision of support for social and emotional issues of both the child and family when necessary. Whilst our home visitors attempted to provide some of this support, it was not the focus of this intervention.

In addition, only the top ten environmental factors were targeted for each family. It may well be that these initial suggestions were just the first step in change and that subsequent visits were needed to focus on other aspects of the home environment to allow a greater intervention effect. Finally, it should be noted that only 36/66 (55 %) caregivers who sought information about this study, agreed to enrol their child. Whilst we had considered home visits to be more convenient for parents, parental anxiety around possible negative judgement about their home and household circumstances may well have contributed to this poor recruitment response.

Trials of home based intervention in other areas of health have had variable success. Segal et al. undertook an extensive review of home visiting for prevention of child maltreatment [29]. They demonstrated that those trials clearly grounded in a theory or mechanism of change and with an intervention most consistently based on this theory were successful. On the other hand, trials where no underlying theory was described were not successful. A positive feature of our current study was that we based the intervention on ecological theory; that is we sought to change the home environments of children with obesity [3, 24]. However, one of the reasons that our intervention may have had limited success, is that ecological theory regarding obesity development suggests that macro-environments exert greater influence on obesity development/maintenance than parent’s ability to optimise the family home environment [30].

In the area of childhood asthma, Brown et al. discussed other possible reasons for the limited benefit observed following regular nurse home visits compared to standard clinic care [31]. Nurses cited poor time management, personal problems or the family’s living environment as the greatest barriers to program success for about 40 % of families. As our study progressed, the complex and sometimes chaotic social issues faced by many families became more obvious and these took priority for families over positive lifestyle change. Brown et al. also reported that nurses who were more judgemental and less flexible were least successful in terms of taking families through completion of the asthma education program [31]. In contrast, the home visitors in our study had been chosen because of their interpersonal style and we had specifically addressed the issue of obesity stigmatisation and importance of not being judgemental in our pre-intervention training.

The findings of this study must be interpreted in the context of its strengths and limitations. This is one of few studies to measure elements of the home environment by direct observation instead of parental report. The study included both assessments of dietary and physical activity patterns rather than focusing on only one side of the energy balance equation. However, as with any self-report methodologies these data could be at risk of measurement and social desirability bias. Practical aspects of ensuring a healthy lifestyle for children by parents were examined resulting in data that has real utility for health professionals dealing with overweight children and their families. However the sample size of this pilot study was small. Results should be viewed with caution and be useful primarily to inform future intervention studies rather than clinical practice.

## Conclusion

In conclusion, this home-based intervention pilot study in which families of overweight and obese children were advised to change aspects of their home environment was not effective in improving children’s dietary and physical activity behaviours, or weight status. Future child obesity interventions should target factors which families struggle to change, provide additional behaviour modification support, and utilise a multi-disciplinary approach to address the complex social circumstances which limit parents’ ability to prioritise healthy family lifestyle behaviours.
